# Adapted approaches to initial fluid management of patients with major burns in resource-limited settings: A systematic review

**DOI:** 10.1016/j.burnso.2024.100365

**Published:** 2024-11

**Authors:** Kai Hsun Hsiao, Joseph Kalanzi, Stuart B Watson, Srinivas Murthy, Ani Movsisyan, Kavita Kothari, Flavio Salio, Pryanka Relan

**Affiliations:** a Emergency Medical Teams, Country Readiness Strengthening Department, World Health Organization, Geneva, Switzerland; b Department of Anaesthesia, Critical Care and Emergency Medicine, College of Health Sciences, Makerere University, Kampala, Uganda; c Canniesburn Plastic Surgery and Burns Unit, Glasgow Royal Infirmary, Glasgow, United Kingdom of Great Britain and Northern Ireland; d Faculty of Medicine, University of British Columbia, Vancouver, Canada; e Consultant to the Methods and Standards Team, World Health Organization, Geneva, Switzerland; f Consultant to Library and Digital Information Networks, World Health Organization, Geneva, Switzerland

**Keywords:** Burns, Disasters, Fluid therapy, Intravenous fluid, Mass casualty incidents, Resource-limited settings

## Abstract

**Background:**

Resource limitations in settings such as burn mass casualty incidents (MCIs) present challenges to the judicious fluid resuscitation required for major burns. Previous recommendations for burns care in MCIs have suggested certain adaptations from routine care, such as delaying intravenous (IV) fluid resuscitation until arrival at facility; using a fluid calculation formula that is independent of percentage of total body surface area (%TBSA) burned; or using fluid calculation formula based on time of arrival to first receiving facility rather than from time of injury, thus omitting ‘catch-up’ fluid.

**Objectives:**

To synthesize and assess certainty of evidence from resource-limited settings on the three adaptations to fluid resuscitation for patients with major burns in MCIs.

**Methods:**

PubMed, EMBASE, CINAHL, and Cochrane Library were searched on 8 September 2023 with an update search on 8 July 2024. Primary quantitative studies in resource-limited settings meeting eligibility criteria as assessed by two reviewers were included. Where available, outcome effects for these adaptations compared to routine burns care were calculated. Evidence certainty was determined by GRADE.

**Results:**

Two eligible studies were identified from 544 search results. One study with 48 participants provided very uncertain evidence that delayed IV fluid resuscitation may increase acute kidney injury compared to prehospital resuscitation (OR 2.48, 95% CI 0.58–10.62). The other study with a cohort of 10 children provided very uncertain evidence that calculating fluid requirements based on time of arrival to first receiving facility, i.e. omitting ‘catch-up’ fluids, may maintain adequate urine output and be associated with no complications of fluid over- or under-resuscitation. There were no studies on use of a simplified %TBSA-independent fluid calculation formula.

**Conclusions:**

There is very limited and uncertain evidence to inform on delayed IV fluid resuscitation, simplified %TBSA-independent formula, and omission of ‘catch up’ fluids for burns care in MCIs. Contextual factors, local values, preferences and feasibility also need to be considered.

## Highlights

• Evidence on adapted fluid management for major burns in MCIs is very limited.
• Advisability of delayed at-hospital initiation of IV fluids in MCIs is uncertain.
• Evidence on omission of catch-up fluid for delayed presentations is uncertain.
• No clinical studies on using simplified 100 ml/kg/24 h formula for fluid requirements.

## 1 Introduction

Judicious fluid resuscitation is critical in the initial management of major burns. The pathophysiologic response to major burns, with systemic inflammation, increased microvascular permeability and intravascular volume depletion, described as burn shock, requires large volume fluid resuscitation to prevent hypoperfusion, end-organ (such as renal) dysfunction, and death [1]. However, excessive fluid administration can also lead to significant complications such as pulmonary edema and abdominal compartment syndrome [1], [2]. Addressing this critical but challenging management in fluid balance are several established tenets in routine burns care: (1) Prompt initiation, ideally from time of burn injury, of intravenous (IV) fluid resuscitation for major burns ≥15–20% total body surface area (TBSA); (2) estimation of initial fluid requirements using set formula, accounting for body weight and burn size; and (3) close monitoring and titration of fluid resuscitation based on defined clinical and/or physiologic end-points, such as maintenance of urine output ≥0.5–1.0 ml/kg/hr in adults and ≥1.0–1.5 ml/kg/hr in children [3], [4], [5], [6], [7].

However, these standard approaches to initial fluid management face additional challenges in situations of mass casualty incidents (MCIs) and other resource-limited settings where demand for resources outstrips supply, such as: delays to patient presentation or access to medical care; delays to initiation of fluid resuscitation; insufficient supplies and/or skilled medical personnel for safe IV fluid administration, monitoring, and titration; degraded clinical handover and tracking of fluid administration between services and levels of care; and inaccuracies in burn size estimates leading to fluid under- or over-resuscitation [8], [9], [10], [11], [12]. Recognizing these challenges, a set of recommendations for burns care in MCIs published in 2020 [10] suggested: (1) IV fluid replacement therapy should not be delivered on scene but at the first receiving hospital after further clinical assessment; (2) a simplified formula of 100 ml/kg/24 h for initial fluid replacement should be used; and (3) administered fluid regimen should be calculated from time of arrival at first receiving facility and not the time of burn, that is, no ‘catch-up’ fluid should be given.

The purpose of this review was to synthesize and assess the certainty of available evidence on these three recommended MCI adaptations to routine burns care. This was part of a process to inform a World Health Organization (WHO) Guideline Development Group (GDG) in their development and update of guidelines for burns care in MCIs for Emergency Medical Teams (EMTs). An earlier iteration of this systematic review by the authors had narrowly focused on the MCI setting and yielded no studies (unpublished, PROSPERO study protocol registration #CRD42023430738). This current review broadened the approach and sought evidence in similar and relevant resource-limited settings.

### 1.1 Review question

The PICO-structured review question, with three nested interventions and comparisons, was: **(Population)** For patients with major burns (≥15% TBSA for adults and ≥10% TBSA for children) in resource-limited settings, does **(Interventions/Comparisons)** (i) delayed initiation of IV fluid therapy until arrival at first receiving facility compared to initiation on scene or during prehospital care, or (ii) use of a simplified, %TBSA-independent formula (100 ml/kg/24 h) for calculating fluid requirements compared to standard %TBSA-dependent formulae, such as Parkland, Brookes, among others, or (iii) calculating fluid requirements from time of arrival at first receiving facility (that is, no ‘catch up’ fluids) compared to standard calculation from time of injury **(Outcomes)** affect the outcomes of mortality, urine output, acute kidney injury (AKI), or complications of fluid over-resuscitation?

## 2 Materials and methods

A study protocol was developed and registered on PROSPERO (registration #CRD42023462855). The review conforms to the Preferred Reporting Items for Systematic Reviews and Meta-Analyses (PRISMA) guidelines [13].

### 2.1 Review question formulation and definitions

The review topic was initially identified by the WHO GDG. Subsequently, the review question was formulated by the authors, and affirmed by the GDG.

The population of interest was patients with burn injury of a severity that required fluid resuscitation (i.e. major burn). The lower-end thresholds that typically define this are TBSA ≥15% in adults and ≥10% in children, as specified in some guidelines [14], [15], [16]. This lower-end definition was adopted for this review to ensure broader capture of relevant studies.

Resource-limited settings were defined as settings in which there are inadequate quantity, quality, or access to the necessary skilled staff, supplies, infrastructure and/or organizational processes to provide or maintain commonly accepted standards of care. In the context of burns care, the described settings in which this may occur are: (1) low- and middle-income countries (LMICs), typically defined by the World Bank country income classifications [17], (2) battlefield/far-forward combat casualty care settings, (3) austere or wilderness environments at distance from usual places of health care; and (4) MCI or disaster scenarios where demand overwhelms existing or residual capacities [9], [18], [19]. For this review, MCI settings were excluded to avoid duplication of the prior MCI-specific review, which had yielded no studies (unpublished, PROSPERO study protocol registration #CRD42023430738).

The outcomes of interest were selected and based on prioritization by the WHO GDG. These had included pulmonary edema and abdominal compartment syndrome as separate outcomes, but were subsequently grouped together under complications of fluid over-resuscitation.

### 2.2 Search strategy

The search strategy was developed and conducted by a health information specialist (KK) on the following databases: PubMed, EMBASE, CINAHL, and Cochrane Library. Search strings were adjusted as appropriate for each database and included the following search terms and appropriate synonyms: burns AND fluid therapy AND (resource-limited OR austere OR wilderness medicine OR military medicine OR developing country) NOT enteral nutrition. No filters or limits were applied. The search was conducted on 8 September 2023 with an update search on 8 July 2024. An example search strategy is provided in Appendix.

Additionally, a Google search using the primary search terms “burn”, “fluid resuscitation” and “resource limited” as well as reference search of identified studies, reviews and relevant published guidelines were conducted.

Search results were exported into the Covidence systematic review program (Veritas Health Innovation Ltd., Melbourne, Australia) where records were deduplicated.

### 2.3 Eligibility criteria and study selection

The pre-defined criteria for inclusion were studies that (1) were primary, empirical human studies with quantitative data, including case reports and case series; (2) had a study population (or identifiable sub-group with corresponding disaggregated data) of patients with major burns (i.e. ≥15% TBSA in adults or ≥10% TBSA in children); (3) were set in a resource-limited setting, defined as an LMIC according to World Bank country income classifications [17], a battlefield/far-forward combat casualty care setting, or an austere or wilderness environment; (4) reported on one or more of the interventions of interest, i.e. timing of initiation of IV fluid therapy, formula used for calculation of fluid therapy, and/or time point used for calculation of fluid therapy; (5) included outcomes in terms of overall mortality, urine output, AKI, or complications of fluid over-resuscitation (such as pulmonary edema and abdominal compartment syndrome); and (6) were written in English.

Studies were excluded if they (1) were editorials, commentaries, study protocols, modelling or animal studies; (2) were not set in one of the defined resource-limited settings; (3) were set in the specific resource-limited setting of an MCI or disaster (to avoid duplication of an already conducted review); (4) did not report on the interventions of interest, i.e. delayed initiation of fluid therapy, simplified %TBSA-independent formula for calculation of fluid therapy, and/or calculation of fluid therapy from time of arrival, i.e. omission of ‘catch up’ fluid; or (5) did not include data on any of the outcomes of mortality, urine output, AKI, or complications of fluid over-resuscitation.

Following removal of duplicates, title and abstract screening and subsequent full-text review were independently conducted by two reviewers (KH and JK) according the above criteria to identify eligible studies. In the event of disagreement, a third reviewer (PR) was available to adjudicate.

### 2.4 Data extraction

Data was extracted on Covidence systematic review program (Veritas Health Innovation Ltd., Melbourne, Australia) using a pre-specified standardized form. For each study, data was extracted on: Study characteristics (author, year, title, study type/design, country and setting); study population (sample size, age, type(s) of burn injury, severity of burn, additional life-threating injuries, time to arrival at first receiving hospital, and sub-groups within study); intervention characteristics (timing of initiation of IV fluid therapy, formula used for fluid calculation, and time point used for fluid calculation); outcomes (mortality rate, urine output, occurrence of AKI, and complications of fluid over-resuscitation). Data was independently extracted by two reviewers (KH and JK) and final consensus achieved through discussion. In the event of disagreement, a third reviewer (PR) was available to adjudicate.

### 2.5 Risk of bias assessment

Risk of bias assessments were independently conducted by two reviewers (KH and JK) using the pre-specified JBI Critical Appraisal Checklist for Cohort Studies [20]; cohort studies were the only type of studies ultimately included in this review. In the event of disagreement, a third reviewer (PR) was available to adjudicate. Risk of bias figures were generated using the Risk of Bias Visualization (robvis) tool (McGuinness, LA, et al., Bristol, UK) [21].

### 2.6 Data synthesis and statistical analysis

Separate analyses were conducted for each of the three comparisons in the review question. Where amenable, intervention effect estimates were summarized using odds ratios (with corresponding 95% confidence intervals (CI)) for dichotomous outcomes (mortality, AKI, and complications of fluid over-resuscitation), and mean differences (with corresponding 95% CI) for continuous outcomes (urine output and serum creatinine levels). Meta-analysis had been intended in the study protocol; however, there were not sufficient suitable studies.

Sub-group analyses by age (<18 years old versus ≥18 years old), by burn type, by burn severity (TBSA <40% versus TBSA ≥40%) and for those with inhalational injuries had been included in the study protocol. These sub-groups were identified by the GDG as clinically important. However, given the paucity of studies, sub-group analyses were not possible.

### 2.7 Certainty of evidence assessment

Certainty of evidence was assessed using the Grading of Recommendations Assessment, Development and Evaluation (GRADE) approach [22]. Two reviewers (KH and JK) independently rated the risk of bias, inconsistency, indirectness, publication bias and imprecision for each comparison and outcome that had effect estimates. In the event of any disagreements that could not be resolved by discussion, a third reviewer (PR) was available to adjudicate.

## 3 Results

**Fig. 1:**
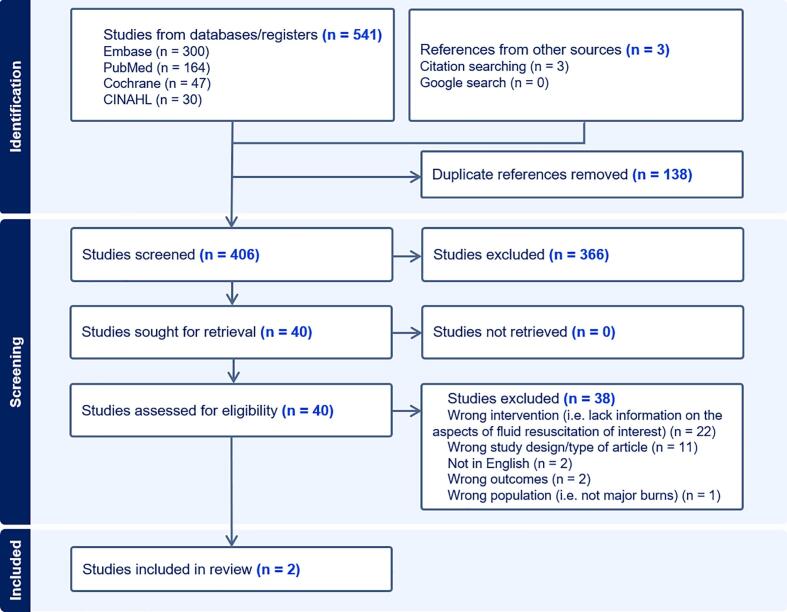
PRISMA flow diagram for study selection.

The search strategy yielded 512 records in the initial search, and an additional 32 records in the update search. Following deduplication, screening, and full-text review, two studies [23], [24] were found eligible for inclusion in this review. Fig. 1 shows the PRISMA flow diagram for study selection, including exclusion reasons.

### 3.1 Study characteristics

Key characteristics of the two included studies are summarized in Table 1. One study [23], which pertained to delayed initiation of IV fluid until receiving facility, was a retrospective cohort study of combat casualties (n=48) with >20% TBSA burns that were directly transported to a combat support hospital in Iraq (battlefield/combat casualty setting). The other study [24], which pertained to calculation of fluid requirements from time of arrival, was a single-armed cohort study of children (n=10) with 15–35% TBSA burns that were admitted to a regional burns care hospital in South Africa (LMIC setting).

**Table 1:** Characteristics of included studies, n=2.

			**Study population**							
**Author, year**	**Setting, country**	**Study design**	**N**	**Age range**	**%TBSA burn range**	**Exclusion criteria**	**Intervention**	**Comparison**	**Reported outcome (s)** **relevant to review**	**Key findings**
Lairet, 2012	Battlefield/ combat casualty, Iraq (Ibn Sina Combat Support Hospital, Baghdad)	Cohort	48	19-41 yrs old Mean: 25 yrs old	>20% (range not reported)	• Non-US casualties • <20% TBSA burns • Non-thermal burns • Casualties transported from another facility	No prehospital IV fluids (n=19) • Mean time to facility: 26.9 min • %TBSA: n/a • Age range: n/a	Prehospital IV fluids (n=15) • Mean time to facility: 43.1 min • %TBSA: 20-80% • Age range: n/a	• AKI	• Trend toward higher incidence of AKI in group with no prehospital IV fluids (9/19, 47.4%) versus group with prehospital IV fluids (4/15, 26.7%), p=0.22
Allorto, 2022	LMIC, South Africa (Edendale Hospital, KwaZulu Natal)	Descriptive cohort (single-arm)	10	2-8 yrs old Mean: 3.8 (SD 2.5) yrs old Median: 3 (IQR 2-5) yrs old	15-35% Mean 21.1% (SD 7.0%) Median: 17.4% (IQR 16-26%)	• Adults • Presentations >24 hrs post-burn injury	Fluids calculated from time of arrival (i.e. no ‘catch up’ fluids) (n=10) • Modified Brookes formula of 2mls/kg per %TBSA divided by 24hrs (irrelevant to the time of burn) for starting resuscitation rate with IV Ringer’s lactate • Preceding 20ml/kg fluid bolus if shocked • Adjusted according to urine output • Mean time to facility: 12.3 hrs (SD 6.5 hrs), range 4-22 hrs	--	• Urine output (adequacy and mean ml/kg/hr during first 24hrs) • AKI • Complication of fluid over-resuscitation	• All (10/10) maintained adequate urine output >0.5 ml/kg/hr (mean 1.66 ml/kg/hr, SD 0.6 ml/kg/kg/hr) in first 24hrs • None (0/10) developed complications of under-resuscitation, including AKI or persistent burn shock • None (0/10) developed complications of over-resuscitation, including pulmonary edema or sodium derangement

LMIC: low- and middle-income countries; SD: standard deviation; IQR: inter-quartile range; %TBSA: percent total body surface area.

### 3.2 Risk of bias within studies

**Fig. 2:**
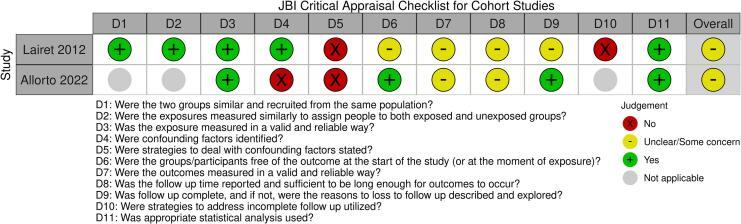
Assessed risk of bias within cohort studies using JBI Critical Appraisal Checklist.

The assessed risk of bias within studies using the JBI Critical Appraisal Checklist for Cohort Studies are presented in Fig. 2. Both studies were assessed to have some concerns for overall risk of bias. For the study by *Lairet et al.* [23], as a retrospective review of medical records, potential incompleteness and errors in the records as well as lack of information to compare baseline characteristics of groups were limitations. For the study by *Allorto et al.* [24], the lack of a comparison group leaves in question alternative possibilities apart from the intervention that may explain the observed outcomes.

### 3.3 Effects of delayed initiation of IV fluid resuscitation until arrival versus prehospital initiation

One study [23] with a total of 48 participants provided comparative effect estimates for combat casualties who received prehospital IV fluid resuscitation versus those who received no prehospital IV fluid. Outcomes were reported only for the subset of burn patients without other trauma and with complete information (n=34).

Higher AKI occurrence was observed with no prehospital fluids (n=9/19) compared to with prehospital fluids (n=4/15), OR 2.48 (95% CI 0.58–10.62), p=0.22.

No data on mortality, urine output or complications of fluid over-resuscitation was reported.

### 3.4 Effects of using simplified %TBSA-independent formula versus standard %TBSA-dependent formula

No eligible studies were found.

### 3.5 Effects of calculating fluid requirements using time of arrival versus using time of injury

The review identified one descriptive, single-arm cohort study [24] that reported the outcomes of 10 children that received initial IV fluid resuscitation on arrival according to 2 ml/kg/%TBSA over 24 h without regard to time of burn, i.e. no ‘catch up’ fluid was given. In this cohort, delays to presentation ranged from 4 to 22 h (mean 12.3 h) post-burn injury. All maintained adequate urine output >0.5 ml/kg/hr during the first 24 h, and none developed complications of fluid under-resuscitation, such as AKI, nor complications of fluid over-resuscitation, such as pulmonary edema. However, the intervention effect of omitting ‘catch up’ fluids compared to including them remains undetermined as there was no comparison group in this study.

### 3.6 Certainty of evidence

Using the GRADE approach, the certainty of evidence of the estimated effect of delayed initiation of IV fluid resuscitation versus prehospital initiation on AKI was rated very low, i.e. very uncertain. From a starting rating of low as an observational study, further downgrading was because of (1) serious risk of bias with some concerns in study limitations; (2) serious indirectness as the findings were based on individual burns care in a combat casualty setting, and although similar to MCI settings in terms of relative resource limitations, differences such as lesser delays to hospital presentation and access to greater resources on arrival at the military hospital are relevant; and (3) serious imprecision as a single study with small sample size and wide confidence interval.

Certainty of evidence on calculation of fluid requirements using time of arrival was also very low. Starting at a low to very low rating as a descriptive, single-arm cohort study, further downgrading was because of serious risk of bias, serious indirectness, and serious imprecision, for similar reasons as above.

## 4 Discussion

This review found a near absence of studies to inform on proposed adaptations to routine IV fluid resuscitation for major burns in resource-limited settings such as MCIs. Delayed, at-hospital initiation of IV fluids may increase AKI occurrence compared to prehospital initiation, but the evidence is very uncertain. Similarly, the evidence is very uncertain on whether calculating fluid requirements from time of arrival rather than time of injury, that is, omitting ‘catch up’ fluid, is associated with maintained urine output and no complications of fluid under- or over-resuscitation. No clinical evidence is available on the effects of using a simplified formula that is not dependent on estimation of %TBSA burn for calculating initial fluid requirements.

The lack and uncertainty of evidence on these specific facets of burns fluid resuscitation in resource-limited settings is worthwhile placed in the broader context of existing variabilities and lack of definitive consensus in many aspects of burns fluid management, even for standard care [4]. While the importance of adequate fluid resuscitation and titration to response are well-accepted general principles, surveys across both high-resource and resource-limited settings have shown wide variations in approach, including initiation threshold(s), formula used, type(s) of fluid, means of monitoring, titration end-points, and perceived protocol accuracy [25], [26], [27]. Further complicating this variability is the recognized need for context-specific adaptations, including considerations of local values, preferences and feasibility, especially in resource-limited settings such as MCIs [24], [28], [29].

Although this review found suggestion of clinical benefit favouring prehospital initiation of IV fluid resuscitation in a resource-limited setting, albeit with very uncertain evidence, there are also contextual and feasibility factors to consider in implementation in burn MCIs. First are the resource implications. On-scene institution of IV fluid resuscitation requires mobilization of skilled personnel and supplies, which may or may not be feasible depending on casualty numbers, severity of burns, location, access, type of incident, and availability of surge staff and supplies. This also needs to be weighed against the resource cost-benefits of a rapid evacuation and transport approach. For example, in the 2001 Volendam café fire in the Netherlands, which resulted in 245 casualties, an on-scene treatment approach was adopted due to difficulties in transport access, which protracted evacuation [30]. However, this involved deployment of over 200 rescue workers to the scene [30]. A second factor for consideration is the on-scene environmental challenges to accurate patient assessment, and timely and safe initiation and monitoring of IV fluid therapy. From reported experiences, challenges such as insufficient lighting, environmental temperature, patient exposure and privacy, and missing prehospital clinical documentation have been described, and inaccuracies in on-scene burn size assessments quantified [11], [30], [31]. Inaccurate on-scene %TBSA burn estimation and therefore fluid requirement calculation, compounded by inadequate titration due to overwhelming casualties, inexperienced responders and lack of monitoring equipment as well as potential delays to hospital assessment and adjustment, have been part of concerns raised against on-scene IV fluid initiation, especially with the risks of fluid over-resuscitation [10], [28]. Reported Australian experience with retrieved burns patients from the 2002 Bali bombings in Indonesia and the 2009 Ashmore Reef vessel explosion off the coast of western Australia suggested that suboptimal prehospital fluid resuscitation may have been beneficial with anecdotally lower rates of airway edema, and of intubation and ventilatory support needs [32], [33]. Prehospital intubation and ventilatory support would have been scarce, and could have hindered medical evacuation with additional resource and time requirements [32], [33]. The consequences of fluid under-resuscitation, however, were not described. Most existing guidelines and recommendations for burns care in MCIs and other austere settings do not specifically comment on whether on-scene IV fluid resuscitation should or should not be instituted, only that oral fluid resuscitation may be considered if the IV route is not possible or is delayed [8], [9], [14], [19].

The inaccuracies in %TBSA burn estimates and challenges to fluid management in MCIs, as described above, were part of the considerations underlying the proposal for a simplified, %TBSA-independent 100 ml/kg/24 h fluid resuscitation formula [10], [28]. We found no clinical studies applying this simplified formula in resource-limited settings to inform evidence. This may in part be due to its novelty. There are other simplified formulas for burn fluid calculation. The US Department of Defense Joint Trauma System uses the simplified ‘Rule of 10′ formula (10 ml/hr x %TBSA for adults, adding 100 ml/hr for each 10 kg above 80 kg weight) [34]. This formula is still based on %TBSA estimation, and limited studies have indicated mathematical and clinical outcome comparability to standard formula [35], [36]. Even with standard formulas, high level evidence and strong consensus on the optimal formula are lacking [4], [28]. Many different formulas are used in guidelines and in practice, with most ranging from 2 ml/kg/%TBSA/24 h (Brooke) to 4 ml/kg/%TBSA/24 h (Parkland) [27], [28]. A study of burns care practices across the African continent found that the majority used the Parkland formula, but other formulas such as the modified Parkland, Brooke and Galveston were also used [27]. Use of formulas conforming to 2–4 ml/kg/%TBSA/24 h have also been reported for several burn MCIs [37], [38], [39]. *Leclerc et al.* [28] numerically compared the fluid volumes that would be given using the 100 ml/kg/24 h simplified formula to that using the standard 2–4 ml/kg/%TBSA/24 h formulas for adults and the Galveston for children. Their study found that fluid volumes were in agreement for adults with 25–50% TBSA burns, but were under-estimated for children, and markedly so for all with >60% TBSA burns [28]. The proposed strength of this simplified formula was its ease of use, identical for both oral and IV fluids, which is to be implemented in the very specific situation of an overwhelming MCI until specialist burns care is available [28]. However, as an intended add-on to the standard formula in everyday use, consideration should also be given to the potential complexities introduced by having two formulas with differential use, challenges of recognizing situations for switching between standard and simplified formula, and possible confusion on the formula in use when transferring care between providers or levels of care, especially if external surge medical teams are also involved. New or changed clinical practices also often require prolonged periods of sustained effort to achieve functional implementation [40], which may be further challenged when its application is possibly infrequent, depending on context, such as for MCIs. Extrapolating from the reported lesson of benefit in maintaining standard clinical processes for staff familiarity during the Australian Ashmore Reef explosion response [32], there may be argument for reinforcing existing local burns protocols and standard formula that are already familiar to local staff rather than superimposing a novel, situation-specific formula for use during an MCI.

The other aspect of fluid formulas encompassed in this review was the calculation of fluid requirements based on time of injury versus time of arrival to the first facility. Standard formulas calculate fluid requirements from time of injury which, in cases of several hours’ delay to fluid initiation, allows for an initial accelerated rate to catch up on the fluid losses that have already occurred since burn injury [3]. However, there is less clarity on whether this is still applicable when delays are significant, such as >8 h, as may occur in MCI and other austere settings [24]. For example, Parkland formula stipulates fluid replacement at 4 ml/kg/%TBSA/24 hrs with half given in the first eight hours after burn injury and the remainder over the following 16 h [41]. So, for a 70 kg person with 50% TBSA burn presenting at 8 h after burn injury, strict adherence to Parkland would require catch-up infusion of 7000 ml as opposed to a starting rate of 583 ml/hr if the calculated 24-hour fluid requirement was simply applied from time of arrival (i.e. omitting ‘catch-up’ fluid). Very uncertain evidence from a single-arm cohort study found by this review suggested that omission of ‘catch-up’ fluid for patients with delayed presentations of 4–22 h post-burn may be associated with no adverse outcomes [24]. However, the study was limited by its small size, pediatric-only population, and lack of larger injuries with the most severe burn being 35% TBSA [24]. Additionally, the protocol used in this study also included provisions for initial 20 ml/kg fluid boluses for shock and protocolized adjustments to fluid rate according to urine output. Therefore, the favourable outcomes observed in this study may be more reflective of a consensus principle that critically underlies all fluid formulas, that is, the formulas only provide a guide to the starting point for fluid resuscitation and rates must be subsequently adjusted according to clinical response, even in MCI and other resource-limited settings [3], [4], [8], [10], [14], [28].

### 4.1 Limitations

As with all systematic reviews, there may have been relevant studies that were missed by the search strategy and study selection. For this review, a key limitation in this regard was the exclusion of articles not written in English. Another potential limitation was restricting the review question to the context of resource-limited settings. This was in part due to considerations of feasibility: The review question was focused on very specific aspects of major burns fluid resuscitation, and an approach without restricting by context, that is, simply searching based on concepts of “burns” and “fluid resuscitation”, would have likely resulted in an inordinate number of records to screen without necessary gains in eligible studies. The specific adaptations to fluid management that were the focus of this review were based on the context-specific challenges and resource constraints of MCIs. Therefore, the assumption was that such deviations from standard care would unlikely be found or studied outside of similar resource-limited settings. Additionally, the focus on resource-limited settings would make the findings more applicable to MCIs. Conversely, the use of the broader resource-limited settings of LMICs, battlefield/far-forward combat casualty care and austere environments still represented a limitation due to indirectness of evidence. In these settings, the focus remains on optimal individual care within the confines of available resources, whereas in MCIs there is an inherent paradigm shift where trade-offs between optimal individual versus optimal collective outcomes are required [28]. Finally, relevant evidence may also be found in other types of studies, such as modelling or non-clinical studies, which were excluded in this review.

In addition to the limitation of very few studies, there were also limitations within studies. While the sole study on omission of ‘catch up’ fluids provided some clinical outcome data, the lack of a comparison group meant that the effect of omitting ‘catch up’ fluids compared to including them could not be determined.

## 5 Conclusions

There is very limited and very uncertain evidence to inform on whether delayed IV fluid resuscitation, whether a simplified %TBSA-independent fluid resuscitation formula, or whether omission of ‘catch up’ fluid in major burn care may or may not be beneficial in resource-limited settings including MCIs. Further clinical research in each of these aspects as applied to resource-limited settings is warranted. In addition, contextual factors such as local values, preferences and feasibility are also relevant, and need to be considered in any implementation.

## Funding sources

This research did not receive any specific grant from funding agencies in the public, commercial, or not-for-profit sectors.

## Declaration of Competing Interest

The authors declare that they have no known competing financial interests or personal relationships that could have appeared to influence the work reported in this paper.

## Figures and Tables

**Table:** 

Database: Pubmed		
URL: https://www.pubmed.gov		
Date Searched: 08.09.2023		
**#**	**Searches**	**Results**
1	“Burns”[Mesh] OR “burn units”[mesh] OR burn[ti] OR burns[ti] OR postburn*[ti] OR thermally-injured[ti] OR thermal-injur*[ti]	68,839
2	“Rehydration Solutions”[mh] OR “Fluid Therapy”[mh] OR “Plasma Volume”[mh] OR “Resuscitation”[Mesh] OR fluid*[tiab] OR hydration[tiab] OR rehydration[tiab] OR resuscitat*[tiab]	761,595
3	#1 AND #2	4,496
4	“HOSPITALS, MILITARY”[MH] OR “MILITARY MEDICINE”[MH] OR “MILITARY NURSING”[MH] OR “Wilderness Medicine”[MH] OR military[tiab] OR humanitarian-setting*[tiab] OR low-resourc*[tiab] OR limited-resourc*[tiab] OR resource-limited[tiab] OR austere*[tiab] OR scanty[tiab] OR “meager resource”[tiab:∼3] OR meager-resource*[tiab] OR slender-resourc*[tiab] OR “slender resources”[tiab:∼3] OR “small resources”[tiab:∼3] OR “lack resources”[tiab:∼3] OR simplified[tiab]	200,124
5	developing-country[tw] OR developing-countries[tw] OR developing-nation[tw] OR developing-nations[tw] OR developing-population[tw] OR developing-populations[tw] OR developing-world[tw] OR less-developed-country[tw] OR less-developed-countries[tw] OR less-developed-nation[tw] OR less-developed-nations[tw] OR less-developed-world[tw] OR lesser-developed-country[tw] OR lesser-developed-countries[tw] OR lesser-developed-nation[tw] OR lesser-developed-nations[tw] OR lesser-developed-world[tw] OR under-developed-country[tw] OR under-developed-countries[tw] OR under-developed-nation[tw] OR under-developed-nations[tw] OR under-developed-world[tw] OR underdeveloped-country[tw] OR underdeveloped-countries[tw] OR underdeveloped-nation[tw] OR underdeveloped-nations[tw] OR underdeveloped-world[tw] OR middle-income-country[tw] OR middle-income-countries[tw] OR middle-income-nation[tw] OR middle-income-nations[tw] OR middle-income-population[tw] OR middle-income-populations[tw] OR low-income-country[tw] OR low-income-countries[tw] OR low-income-nation[tw] OR low-income-nations[tw] OR low-income-population[tw] OR low-income-populations[tw] OR lower-income-country[tw] OR lower-income-countries[tw] OR lower-income-nation[tw] OR lower-income-nations[tw] OR lower-income-population[tw] OR lower-income-populations[tw] OR underserved-country[tw] OR underserved-countries[tw] OR underserved-nation[tw] OR underserved-nations[tw] OR underserved-population[tw] OR underserved-populations[tw] OR under-served-population[tw] OR under-served-populations[tw] OR under-served-nation[tw] OR under-served-nations[tw] OR under-served-population[tw] OR under-served-populations[tw] OR deprived-country[tw] OR deprived-countries[tw] OR deprived-population[tw] OR deprived-populations[tw] OR high-burden-countr*[tw] OR high-burden-nation*[tw] OR countdown-countr*[tw] OR countdown-nation*[tw] OR poor-country[tw] OR poor-countries[tw] OR poor-nation[tw] OR poor-nations[tw] OR poor-population[tw] OR poor-populations[tw] OR poor-world[tw] OR poorer-country[tw] OR poorer-countries[tw] OR poorer-nation[tw] OR poorer-nations[tw] OR poorer-population[tw] OR poorer-populations[tw] OR poorer-world[tw] OR developing-economy[tw] OR developing-economies[tw] OR less-developed-economy[tw] OR less-developed-economies[tw] OR underdeveloped-economy[tw] OR underdeveloped-economies[tw] OR under-developed-economy[tw] OR under-developed-economies[tw] OR middle-income-economy[tw] OR middle-income-economies[tw] OR low-income-economy[tw] OR low-income-economies[tw] OR lower-income-economy[tw] OR lower-income-economies[tw] OR low-gdp[tw] OR low-gnp[tw] OR low-gross-domestic[tw] OR low-gross-national[tw] OR lower-gdp[tw] OR lower-gnp[tw] OR lower-gross-domestic[tw] OR lower-gross-national[tw] OR lmic[tw] OR lmics[tw] OR third-world[tw] OR lami-country[tw] OR lami-countries[tw] OR transitional-country[tw] OR transitional-countries[tw] OR emerging-economy[tw] OR emerging-economies[tw] OR emerging-nation[tw] OR emerging-nations[tw]	202,325
6	#4 OR #5	392,428
7	#6 AND #3	158
8	enteral-nutrition*[ti] OR feeding[ti]	59,367
9	#7 NOT #8	157
